# NKG2D triggering hampers DNAM-1-mediated signaling in human NK cells

**DOI:** 10.3389/fimmu.2025.1575059

**Published:** 2025-05-12

**Authors:** Caterina Marangio, Nadia Domenica Milito, Erisa Putro, Alessia Carnevale, Cristina Capuano, Alessandra Zingoni, Marco Cippitelli, Angela Santoni, Rossella Paolini, Rosa Molfetta

**Affiliations:** ^1^ Laboratory Affiliated to Istituto Pasteur Italia-Fondazione Cenci Bolognetti, Department of Molecular Medicine, Sapienza University of Rome, Rome, Italy; ^2^ Departmental Faculty of Medicine and Surgery, UniCamillus-Saint Camillus International University of Health and Medical Sciences, Rome, Italy; ^3^ Istituto di Ricovero e Cura a Carattere Scientifico (IRCCS), Neuromed, Pozzilli, Isernia, Italy

**Keywords:** innate immunity, NK cells, NK cell receptors, functional exhaustion, signaling

## Abstract

**Introduction:**

Natural Killer (NK) cells are cytotoxic innate lymphocytes able to detect transformed cells through the balanced action of inhibitory and activating receptors. NKG2D is one of the main activating receptors involved in tumor surveillance thanks to its ability to recognize stress-induced ligands. Of note, the prolonged exposure to NKG2D ligands promotes receptor down-modulation that results in defective activation of NKG2D and other unrelated activating receptors, including DNAM-1 that is also involved in tumor clearance. However, further investigations are necessary to characterize how the NKG2D/DNAM-1 interplay affects NK cell anti-tumor function.

**Methods:**

Primary cultured human NK cells were stimulated with the natural ligand MICA or an anti-NKG2D agonist antibody. The expression of activating and inhibitory receptors as well as DNAM-1-triggered signaling events and cytotoxicity were evaluated by flow cytometry. DNAM-1-mediated granule polarization was evaluated by confocal microscopy.

**Results:**

We showed that NKG2D crosslinking mediated by the natural ligand MICA or an agonist antibody had different consequences on primary cultured human NK cells. Indeed, MICA stimulation increases the expression of the checkpoint receptor TIGIT that is able to counteract DNAM-1 activation. Stimulation with the agonist antibody, without altering TIGIT expression, directly inhibits DNAM-1-mediated signal transduction and cytotoxic function with a mechanism that required NKG2D endocytosis.

**Discussion:**

Our findings contribute to shed light on the functional consequences of NKG2D engagement, demonstrating that a direct impact on DNAM-1-mediated signal transduction occurs independently from the modality of NKG2D crosslinking. Understanding the molecular mechanisms responsible for suppression of NK cell activation may help the development of therapeutic anti-cancer strategies aimed to prevent NK cell dysfunction or to reinvigorate an impaired cytotoxic activity.

## 1 Introduction

Natural Killer (NK) cells are a subpopulation of cytotoxic innate lymphoid cells exerting a fundamental function in cancerous cell elimination as demonstrated by the prognostic significance of their infiltration in tumors (1, 2) and the increasing effort to employ NK cell-based immunotherapies for cancer management (3, 4).

NK cells can discriminate transformed from healthy cells through the coordinated action of inhibitory receptors for major histocompatibility complex class-I (MHC class I) molecules and activating receptors recognizing stress-induced ligands (5–7). Downmodulation of MHC class I molecules frequently occurs during tumor progression and controls NK cell activation through a ‘missing-self’ mechanism. Moreover, the upregulation of stress-induced ligands on cells undergoing malignant transformation provides the ‘induced-self’ activation of cytotoxicity and cytokine release, which contribute to tumor cell clearance (8, 9).

One of the main activating receptors with a compelling role in fighting tumor spreading and progression is the Natural-Killer receptor group 2, member D (NKG2D). In humans, NKG2D recognizes ligands like MHC class I polypeptide-related sequence A and B (MICA, MICB) and UL16 binding proteins 1-6 (ULBP1-6), that are absent or poorly expressed on healthy cells and upregulated on abnormal cells (10, 11).

Ligand-mediated NKG2D engagement triggers granule exocytosis and IFNγ release through a non-covalent association with the transmembrane adaptor DNAX-activating protein of 10 kDa (DAP10) (12) which propagates intracellular signals recruiting the phosphatidylinositol 3-kinase (PI3K) and the complex between growth factor receptor–bound protein 2 (Grb2) and the guanine nucleotide exchange factor Vav1. These molecular complexes promote the phosphorylation of Src homology 2 domain-containing leukocyte protein of 76 kD (SLP-76) and the activation of phospholipase C gamma (PLCγ2) (13, 14). Of note, in freshly isolated human NK cells, the selective engagement of NKG2D engagement can elicit a full activation only in the presence of a pre-activation mediated by cytokines such as IL-2 or IL-15 or when at least another activating receptor is synergistic engaged (15–17).

Ligand binding is also rapidly followed by the internalization of NKG2D/DAP10 receptor complex and its sorting into degradative compartments (18–20).

Upon a persistent stimulation with membrane-bound or soluble ligands, a reduced NKG2D expression was observed on both murine and human NK cells with a consequent impairment of NKG2D-mediated effector functions (19, 21–33).

Of note, accumulating evidence suggest that the chronic NKG2D stimulation is directly linked to the acquisition of an NK cell hypofunctional phenotype (34, 35). This dysfunction is a typical hallmark of cancer progression and is mainly characterized by the downmodulation of cytolytic granule content (36–39) and the up-regulation of inhibitory checkpoint receptors (40–43).

Accordingly, in tumor microenvironment (TME) the prolonged exposure to NKG2D ligands (NKG2DLs) is not only responsible for the defective killing of NKG2DL-positive transformed cells but also affects additional unrelated activating receptors (22, 23, 44–46).

In human NK cells, we have recently demonstrated that persistent NKG2D stimulation with membrane-bound MICA is responsible for the functional impairment of DNAM-1 (46), an unrelated activating receptor that is also able to promote tumor clearance (47, 48). Interestingly, DNAM-1 shares its ligands, PVR and Nectin-2, with the immune checkpoint receptors TIGIT and CD96, that can counteract DNAM-1 function in advanced tumor stages (49–52).

In this paper, we have further investigated NKG2D/DNAM-1 interplay and demonstrate that NKG2D engagement is sufficient to hamper DNAM-1-mediated signal transduction and NK cell cytotoxic function. The impairment of DNAM-1 functionality requires NKG2D endocytosis. Moreover, we show that compared to agonist antibody-mediated NKG2D engagement, the natural ligand MICA is also able to up-regulate the expression of the checkpoint receptor TIGIT.

## 2 Materials and methods

### 2.1 Cell lines and NK cell cultures

Primary human NK cell cultures were obtained by coculturing peripheral blood mononuclear cells (PBMCs) obtained from healthy donors with the irradiated EBV-transformed B-cell line RPMI 8866 for 10 days at 37°C in a humidified 5% CO_2_ atmosphere, as previously described (20). On day 10, the cell population was routinely 85 to 95% CD56^+^, CD16^+^, and CD3^−^ as assessed by flow cytometric analysis. In the experiment of real time PCR, CD3^+^ contaminant T cells were depleted using immunomagnetic beads (Dynabeads CD3, Thermo Scientific; Catalog No: 11151D).

NKL cell line transfected with wild-type DAP10 (DAP10WT NKL cells) or a mutated DAP10 protein in the intracellular Lysine (DAP10K84R), were previous described (20). They were cultured in RPMI 1640 medium supplemented with 10% fetal calf serum (FCS) and recombinant human IL-2 (200 U/ml; Peprotech; Catalog No: 200-02). Ba/F3- PVR were provided by Prof. A. Soriani and generated starting from the cell line Ba/F3, an IL-3-dependent murine pro-B cell line (kindly provided by L.L. Lanier) that were then transfected with pEF6 vector carrying the cDNA for PVR (kindly provided by M. Colonna) as previously described (46) and grown in RPMI 1640 medium supplemented with 10% FCS.

All cell lines were kept in culture for less than two consecutive months and periodically tested for mycoplasma contamination by PCR Mycoplasma Detection Kit (Applied Biological Materials; Catalog No. G238).

### 2.2 *In vitro* stimulation

Ligand stimulation of primary human NK cells was performed adsorbing 2 μg/ml of recombinant MICA Fc-chimera (R&D Systems; Catalog No: 1300-MA) to 96-well plates at 4°C overnight. The same concentration of albumin (BSA) was used as control. Antibody-mediated stimulation was performed adsorbing goat anti-mouse IgG F(ab’)2 fragment (GAM, Jackson Laboratories; Catalog No: 115-006-003) secondary antibody to 96-well plates (5μg/ml) at 4°C overnight. Afterwards, plates were washed twice in PBS and coated with 5 μg/ml of anti-NKG2D (R&D Systems; Clone: 149810; Catalog No: MAB139) for 2 hours at 37°C before adding NK cells (1x10^6^cells/ml). GAM alone was used as internal control. Upon 18 hours of stimulation, NK cells were harvested and used for further analyses.

### 2.3 Flow cytometry

The surface expression of NK cell receptors was analyzed by using anti-NKG2D-PE (Clone: 149810; Catalog No: FAB139P) purchased from R&D Systems, anti-TIGIT-APC (Clone A15153G; Catalog No: 372706), anti-PD1-BV450 (Clone EH12.1; Catalog No: 562516), anti-Tim3-FITC (Clone 7D3; Catalog No: 565568), anti-FasL-PE (Clone: NOK-1 Catalog No: 564261), anti-TRAIL-BV450 (Clone: RIK-2 Catalog No: 564243) from BD Biosciences, anti-CD96-BV450 (Clone: NK92.39; Catalog No: 338417) from BioLegend. DNAM-1 (Bio-Rad; Clone: DX11; Catalog No: MCA2257), and LFA-1 (TS1/18, generous gift of Dr. F. Sanchez-Madrid) were analyzed using unconjugated mAbs followed by GAM-APC Ab (Jackson Laboratories; Catalog No: 115-136-072). To evaluate the expression of cytolytic mediators, NK cells were stained with anti-Perforin-BV450 (BD Biosciences; Clone: δG9; Catalog No: 563393) and anti-GranzymeB-FITC (BD Biosciences; Clone: GB11; Catalog No: 561142) mAbs after fixation and permeabilization with Cytofix/Cytoperm kit (BD Biosciences; Catalog No: 554714). Samples were acquired using a FACSCanto II (BD Bioscience). The MFI subtracted from the MFI of the isotype control antibody was measured using FlowJo software (Ashland, OR) and used to calculate fold changes. Data analysis was performed using FLowJo software v10.8.1.

### 2.4 Functional assays

NK cell degranulation was determined by detection of the cell surface expression of the lysosomal marker CD107a. Recombinant MICA Fc-chimera (2 μg/ml) or anti-NKG2D (5 μg/ml) +GAM (5 μg/ml) were plate-adsorbed, and NK cells were incubated in the presence of APC-conjugated anti-CD107a mAb (BD Biosciences; Clone: H4A3; Catalog No: 641581) and 50μM Monensin (Sigma-Aldrich; Catalog No: M5273). After 4 hours of incubation, cells were harvested and stained for FITC–conjugated anti-CD56 mAb (BD Biosciences; Clone: NCAM16.2; Catalog No: 340410) and analysed by FACSCantoII (BD Biosciences).

NK cell cytotoxic ability was performed as previously described (33). Briefly, primary NK cells were incubated with 2.5μM of carboxyfluorescein diacetate succinimidyl ester (CFSE) (Merck; Catalog No: SCT110)-labelled Ba/F3-PVR at Effector: Target (E:T) ratio ranging from 50:1 to 6:1 for 4 h at 37°C. After washing with PBS/1% BSA, cells were resuspended in PBS/1% BSA and the intercalating DNA dye 7-Aminoactinomycin D (7-AAD) (Merck; Catalog No: SML1633) was added for 20 min at 4°C at the concentration of 5 μg/ml. Cells were then fixed with 1% PFA for 20 min at 4°C and at least 20,000 events in the CFSE+gate were collected using FACSCanto II (BD Bioscience). Data analysis was performed using FLowJo software.

### 2.5 Phosphorylation analyses

Primary cultured NK cells were stimulated with anti-NKG2D mAb, as described earlier. Then, cells were harvested and incubated for 20 min at 4°C with a saturating dose of anti-DNAM-1, followed by GAM at 37°C. The control group was incubated with isotype-matched mAb for 20 min at 37°C, as previously described (53). Finally, cells were fixed and permeabilized with Intracellular Fixation & Permeabilization Buffer Set commercial kit (# 00-5523-00, eBioscience, Thermo Fisher Scientific) according to manufacturer’s instructions, and stained with anti-pAKT-647 (pS473) eBioscience, Thermo Fisher Scientific; Clone: M89-61; Catalog No: 561670) and anti-pERK1/2 (pT202/pY204) (eBioscience, Thermo Fisher Scientific; Clone: G263-7; Catalog No: 558530). Samples were acquired using a FACSCanto II (BD Bioscience) and analysed using FlowJo software (Ashland, OR). Fold change was calculated for each time point as ratio between the MFI of stimulated (anti-DNAM-1+GAM) and unstimulated (Ctrl-Ig+GAM) samples.

### 2.6 RNA isolation, RT-PCR, and real-time PCR

Total RNA was extracted using RNA extraction kit from Genaid Biotech Ltd according to manufacturer’s instructions. RNA integrity and concentration were assessed using themAgilent RNA 6000 Nano Kit and Agilent 2100 Bioanalyzer (Agilent, Santa Clara, CA). Total RNA (0.5 μg) was used for cDNA first-strand synthesis according to the manufacturer’s protocol for murine leukemia virus reverse transcriptase (Promega, Madison, WI). Real-Time PCR was performed using the ABI Prism7900 Sequence Detection system (Applied Biosystems, Foster City, CA). cDNAs were amplified in triplicate with primers for TIGIT (Hs00545087_m1) and GAPDH (Hs99999905_m1) both conjugated with fluorocrome FAM (Applied Biosystems) and fold change was calculated as previously described (46).

### 2.7 Immunofluorescence and confocal microscopy

NKL cells or primary cultured NK cells were stimulated with anti-NKG2D mAb, as described earlier. Then, they were co-cultured with Ba/F3 transfectants for 30 minutes at 37°C, gently resuspended, and plated on poly-L-lysine-coated multichambered glass plates. Cells were then fixed, permeabilized and stained with the anti-Perforin mAb (Ancell; Clone: δG9; Catalog No: 358-020) followed by AlexaFluor594-conjugated GAM-IgG2b (Thermo Scientific; Catalog No: 11005) and AlexaFluor488-conjugated Phalloidin (Thermo Scientific; Catalog No: 12379) diluted in blocking buffer (0.01% Triton-X-100, 1% FCS). After extensive washing, cover slips were mounted using SlowFade Gold reagent with DAPI (Thermo Scientific; Catalog No: S36938) and acquired as previously described (46). In experiments with NK transfectants, samples were acquired using Zeiss LSM 980 confocal microscope with a Plan-Apochromat 63x/1.40 NA oil immersion objective (all from Zeiss – Jena Germany). Images were processed with ZEISS ZEN 3.7 Software as previously described (54). Perforin polarization was evaluated calculating the number of conjugates containing at least 70% of granules localized in the immunological synapse area as previously described (55).

### 2.8 Cell lysis and western blotting

Cells were lysed in a buffer (pH 7.5) containing 1% Triton X-100, 50 mM tris-HCl, 150 mM NaCl, 2.5 mM EGTA, 2.5 mM EDTA, 1.5 mM MgCl2, 1 mM PMSF, 1 mM Na3VO4, 5 mM NaF, 10 mg/ml aprotinin, and 5 mg/ml leupeptin. Total cell lysates were resolved by SDS-PAGE and transferred to nitrocellulose filters. After undergoing blocking of nonspecific reactivity, filters were incubated with anti-Granzyme B (Cell Signaling Technology; Clone: D6E9W; Catalog No: 46890), anti-Perforin (Cell Signaling Technology; Clone: E7D8R; Catalog No: 62550) and anti-β-actin (Sigma-Aldrich; Clone: AC-15; Catalog No: A5441) as primary antibodies followed by peroxidase labelled anti-rabbit IgG (Cytiva Lifescience, Catalog No: NA934) or anti-mouse IgG (Cytiva Lifescience, Catalog No: NA931) secondary antibodies. After extensive washing, the immunoreactive bands were detected with an enhanced chemiluminescence detection kit (Cynagen, Westar ηC Ultra 2.0; Catalog No: XLS075) and exposed to Invitrogen iBrightTM Imaging Systems (Thermo Scientific).

### 2.9 Statistical analysis

Statistical significance between two groups was determined by performing two-tailed, paired Student’s t-test. Differences between multiple groups were analyzed with one-way or two-way ANOVA, as indicated. Prism 10 (GraphPad) software was used. Graphs show mean values and all error bars represent the SD.

## 3 Results

### 3.1 NKG2D engagement by plate-bound ligand or an agonist antibody differently affects TIGIT expression

By employing transfectants overexpressing the NKG2D ligand MICA, we have previously demonstrated that sustained NKG2D stimulation impairs DNAM-1 functionality through two independent mechanisms: the up-regulation of TIGIT expression that competes with DNAM-1 for ligand binding, and a concomitant direct impairment of DNAM-1-triggered signal transduction (46). However, further investigations are necessary to better dissect the pathways of DNAM-1/TIGIT balance that affects NK cell anti-tumor function.

**Figure 1:**
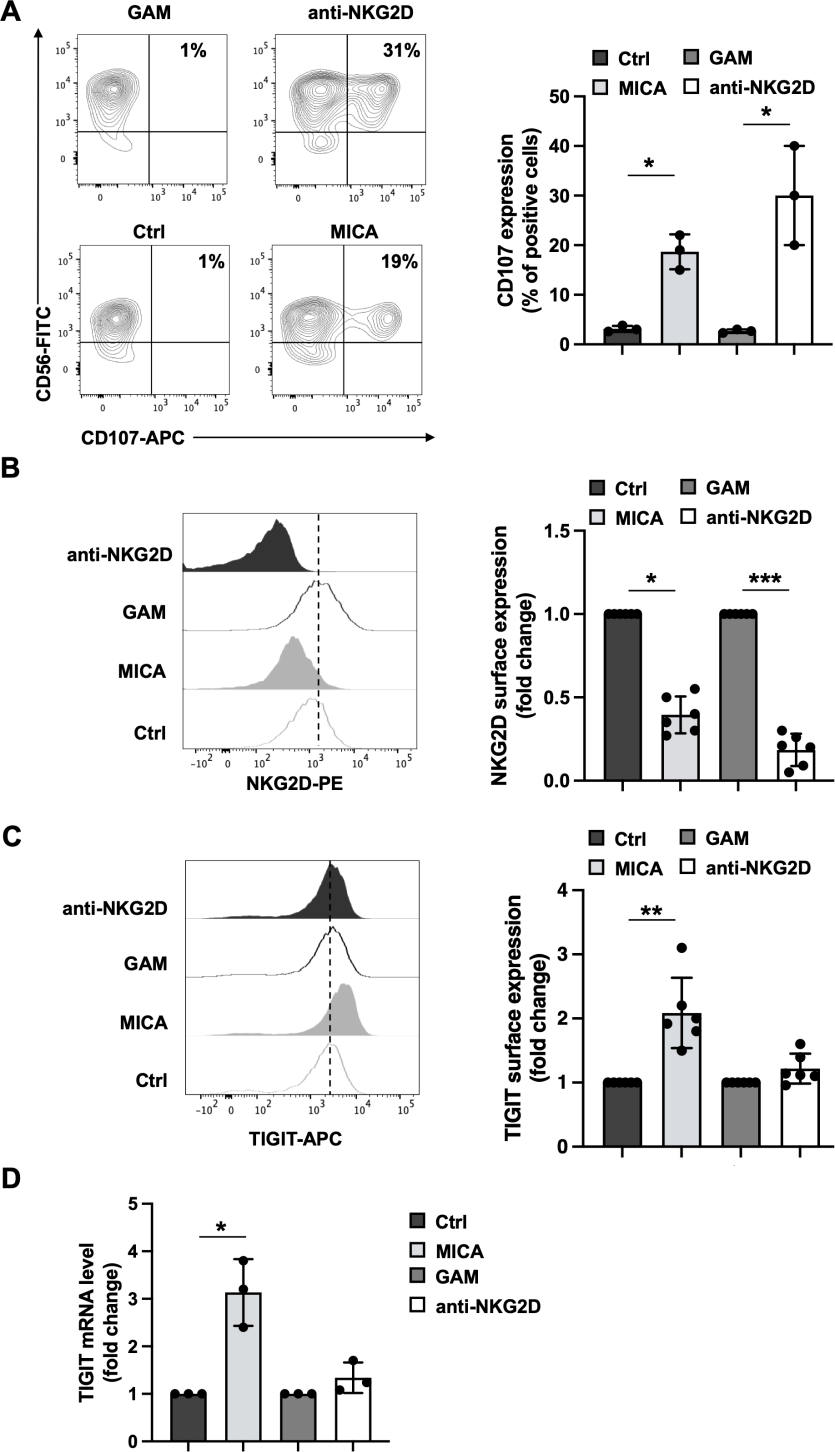
Consequences of MICA-mediated and antibody-mediated NKG2D engagement on cell activation, NKG2D and TIGIT expression. Primary cultured NK cells were incubated with plate-bound recombinant MICA Fc-chimera or anti-NKG2D mAb and goat anti-mouse antibody (GAM). GAM-stimulated NK cells were used as control of NKG2D stimulation. **(A)** Cells were harvested after 4 hours of stimulation, and degranulation was measured via flow cytometry by assessing CD107a endosomal marker expression. A representative experiment is shown in left panel while means ± SD of three independent experiments are shown in right panel. Paired t test was used for comparisons. *P < 0.05. **(B, C)** Surface expression of NKG2D and TIGIT receptors was evaluated by FACS analysis upon 18 hours of stimulation. One representative experiment is shown in left panels. Right panels: data pooled from six independent experiments (mean ± SD) are shown (the mean fluorescence intensity, MFI, value of controls NK cells in each experiment is set to 1). Paired t test was used for comparisons. *ρ < 0.05, **ρ < 0.01, ***ρ < 0.001. **(D)** Relative TIGIT mRNA amount, normalized with GAPDH, was expressed as arbitrary units and referred to the relative controls, considered as calibrator (the value of the calibrator in each run is set to 1). Means ± SD of five independent experiments are shown. Paired t test was used for comparisons. *ρ < 0.05.

To exclude the possible contribution of additional ligand/receptor interactions in altering DNAM-1/TIGIT balance, we assayed the impact of individual NKG2D crosslinking. Primary cultured NK cells were incubated for 4 hours with plate-bound MICA recombinant protein or, alternatively, plate-bound anti-NKG2D monoclonal antibody (mAb). Compared to their respective controls, both stimuli promoted NK cell granule release, evaluated by the induction of CD107a surface expression by flow cytometry (**Figure 1A**). To analyse the impact of a more prolonged stimulation, primary cultured NK cells were challenged for 18 hours in the presence of plate-bound recombinant MICA or anti-NKG2D mAb. NKG2D and TIGIT expression was evaluated by flow cytometry gating CD56^+^ cells as shown in **Supplementary Figure S1**. NKG2D internalization, shown as a decrease in NKG2D surface expression (**Figure 1B**), was observed in both stimulation conditions. As expected, MICA was also able to upregulate TIGIT membrane expression (**Figure 1C**) as well as its mRNA levels (**Figure 1D**). However, the expression of TIGIT and its mRNA remained unaltered upon stimulation with anti-NKG2D mAb (**Figures 1C, D**).

We then evaluated whether the use of agonist antibodies specific for the activating receptors NKp46 and 2B4 could also induce TIGIT expression. We found that only the anti-NKp46 mAb was able to strongly increase TIGIT expression either alone or upon NKG2D co-stimulation (**Supplementary Figure S2**).

These data indicate that both stimuli are able to induce NKG2D endocytosis and lytic granule release but only MICA-mediated NKG2D crosslinking is sufficient to trigger TIGIT upregulation. On the other hand, antibody-mediated NKG2D engagement does not increase TIGIT expression that occur only with the co-stimulation with other unrelated activating receptors (**Supplementary Figure S2**).

### 3.2 Antibody-mediated NKG2D stimulation impairs DNAM-1 intracellular signals and cytotoxicity

**Figure 2:**
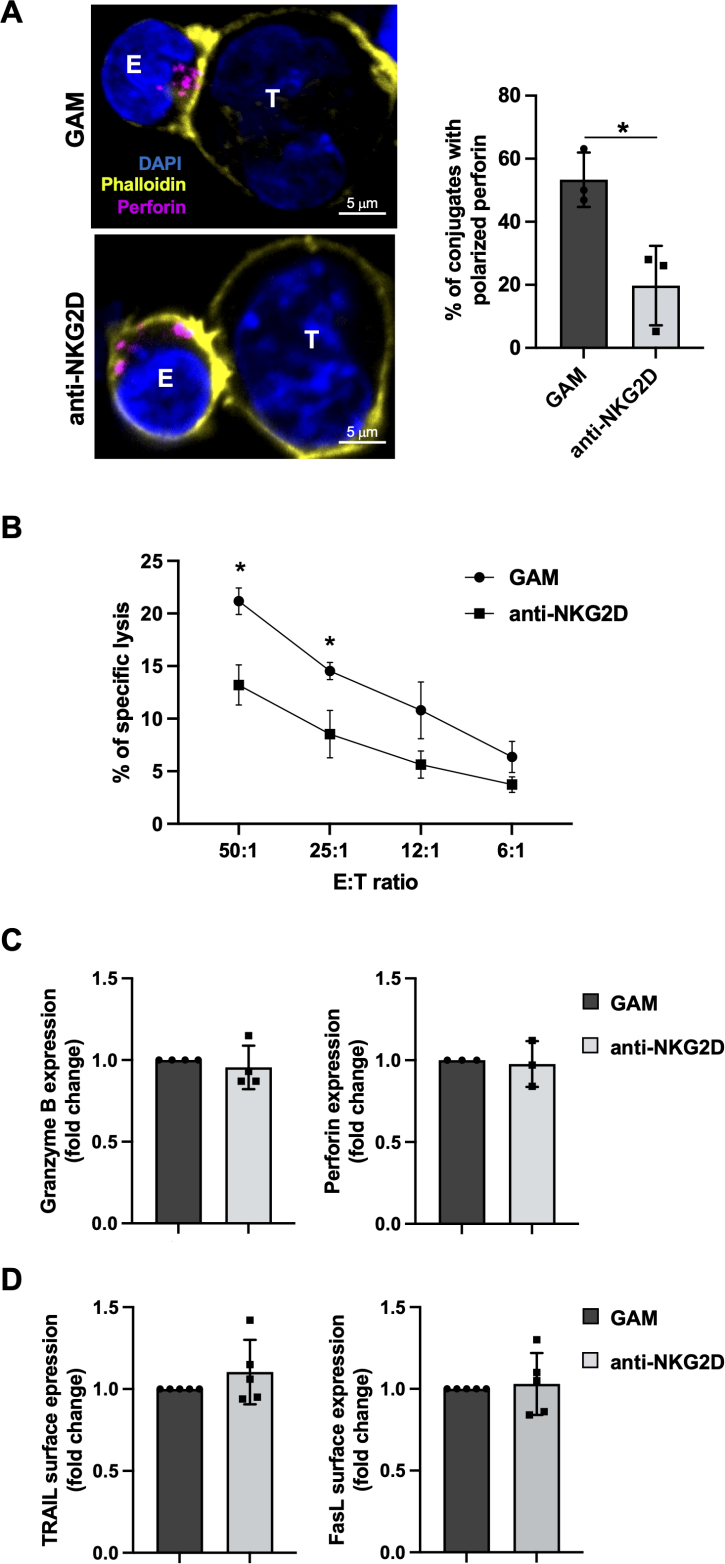
Antibody-mediated NKG2D stimulation impairs DNAM-1 cytotoxicity without inducing NK cell exhaustion **(A)** Left panel: goat anti-mouse (GAM) or anti-NKG2D-stimulated NK cells (effector cells, E) were incubated with Ba/F3-PVR (target cells, T) for 30 minutes, fixed, permeabilized and stained with the anti-Perforin mAb followed by AlexaFluor594-conjugated GAM-IgG2b and AlexaFluor488-conjugated Phalloidin to visualize polarized actin. Cells were then counterstained with DAPI to visualize nuclei and acquired with IX83 FV1200 MPE laser scanning confocal microscope with a 60x/1.35 NA UPlanSAPO oil immersion objective (all from Olympus). Right panel: Percentages of conjugates with polarized granules are shown as mean ± SD calculated on ≥30 conjugates for each NK cell type of four independent experiments. Paired t test was used for comparisons. *ρ < 0.05. **(B)** goat anti-mouse GAM and anti-NKG2D-stimulated primary NK cells were used as effectors in a flow cytometry-based cytotoxicity assay against Ba/F3-PVR. Percentage of target cell death was evaluated by staining with the intercalating DNA dye 7-Aminoactinomycin D (7-AAD) at different effector:target (E:T) ratios, as indicated. Mean ± SD of three independent experiments is shown. One-way ANOVA was used for comparisons. *ρ < 0.05. **(C, D)** Intracellular expression of Granzyme B and Perforin **(C)** and surface expression of TRAIL and FasL **(D)** were evaluated on GAM and anti-NKG2D-stimulated NK cells by FACS analysis. Data pooled from five independent experiments (mean ± SD) are shown (the MFI value of GAM-stimulated NK cells in each experiment is set to 1).

We then decided to investigate whether DNAM-1 functionality is affected upon antibody-mediated NKG2D stimulation even in the absence of TIGIT upregulation. To this purpose, upon an overnight stimulation with anti-NKG2D mAb, NK cells were recovered and co-cultured with PVR overexpressing cells (Ba/F3-PVR). Lytic granule polarization towards target cells was evaluated by confocal microscopy using anti-Perforin staining while target cell death was evaluated by a flow cytometry-based assay using the intercalating DNA dye 7-Aminoactinomycin D (7-AAD). Compared to cells control cells (GAM alone), NKG2D-stimulated NK cells resulted less able to polarize lytic granules (**Figure 2A**) and to kill PVR-expressing targets (**Figure 2B**), supporting the existence of a TIGIT-independent impairment of DNAM-1 cytotoxicity.

Among features that characterize NK cell exhaustion in TME, decreased levels of lytic granule content as well as ligands for death receptors, including FasL and TRAIL, have been demonstrated (36–39). Therefore, we analyzed whether antibody-mediated NKG2D engagement induces a general impairment of NK cell cytotoxic functions. Flow cytometric analysis and western blotting showed that protein level of Granzyme B and Perforin do not decrease upon overnight NKG2D stimulation (**Figure 2C**; **Supplementary Figure S3**). Moreover, death membrane receptor expression remained unaltered as evaluated by flow cytometry (**Figure 2D**).

Thus, NKG2D engagement results in DNAM-1 hypo-functionality without promoting an exhausted phenotype. It is likely that a more prolonged stimulation is necessary to observe a profound dysfunction, as recently reported (35).

**Figure 3:**
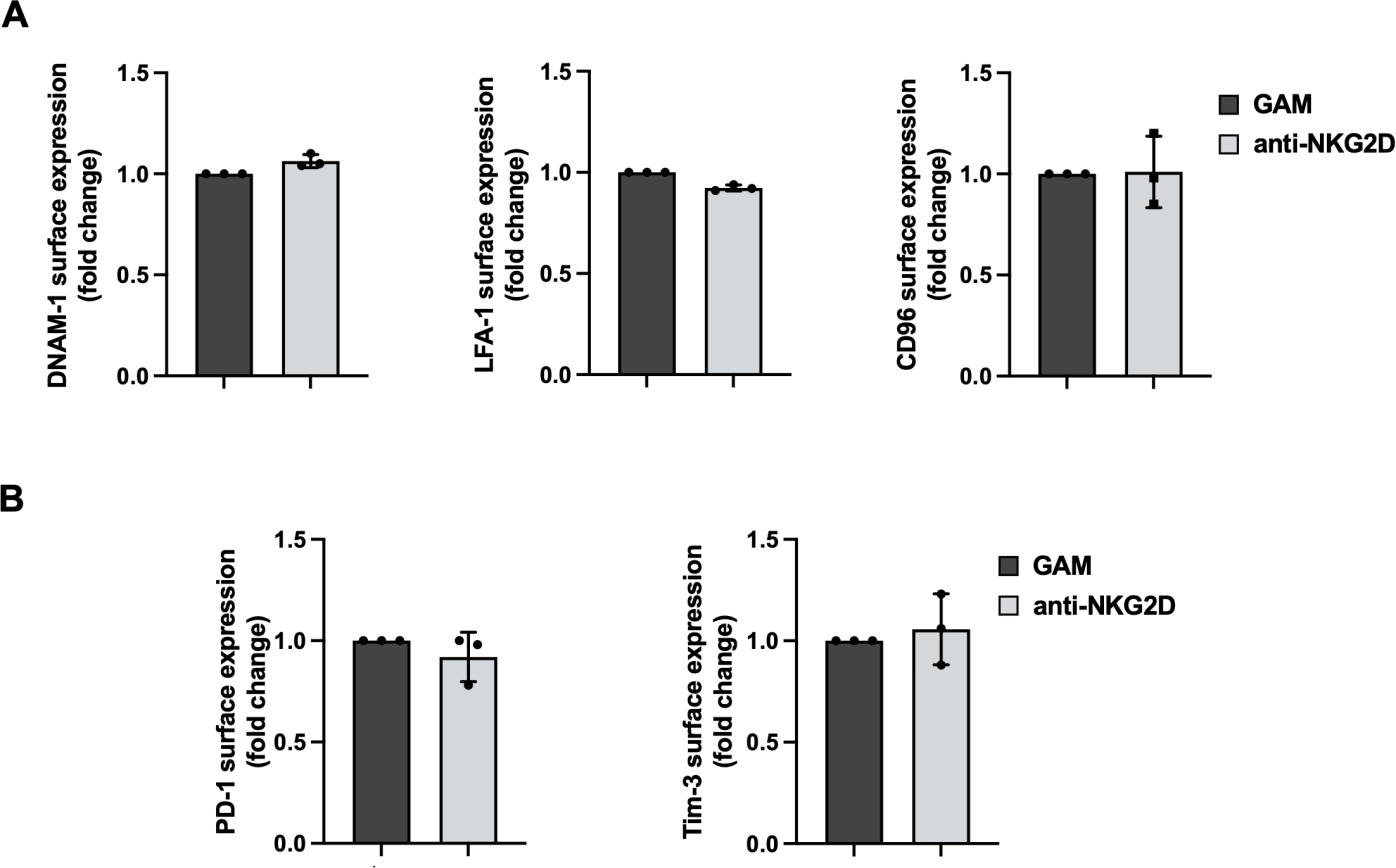
Anti-NKG2D stimulation does not alter activating and inhibitory receptor expression. **(A, B)** Surface expression of DNAM-1, LFA-1, CD96 **(A)**, PD-1 and Tim-3 **(B)** were evaluated on goat anti-mouse (GAM) and anti-NKG2D-stimulated NK cells by FACS analysis. Data pooled from three independent experiments (mean ± SD) are shown (the MFI value of GAM-stimulated NK cells in each experiment is set to 1).

We then evaluated by flow cytometry whether the decreased ability to kill PVR-expressing targets was due to changes in surface expression levels of activating and inhibitory receptors that can affect DNAM-1 activation. Surface expression of DNAM-1, the functionally related LFA-1, and CD96 were all unaltered (**Figure 3A**). Moreover, unrelated checkpoint receptors such as PD-1 and Tim3 remained untouched (**Figure 3B**).

Since NK cell killing ability against PVR-expressing targets is strongly reduced even in the absence of TIGIT up-regulation (**Figure 2**), we investigated whether NKG2D stimulation promotes a defective DNAM-1-mediated signal transduction.

Intracellular events triggered by DNAM-1 crosslinking involve the recruitment of the Grb2/Vav1 adaptor complex which allows the activation of PI3K/AKT pathway and ERK1/2 phosphorylation (56).

**Figure 4:**
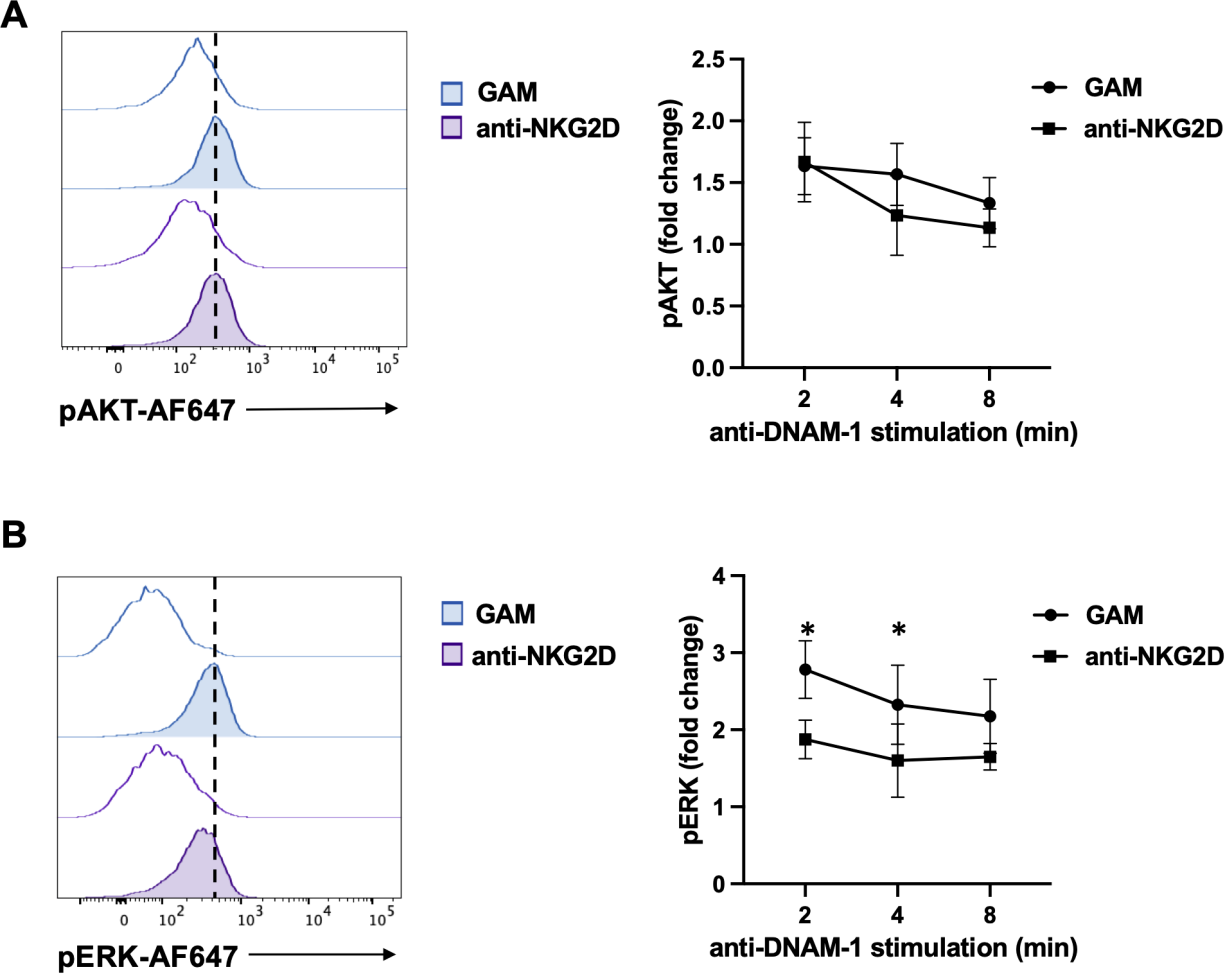
NKG2D stimulation impairs DNAM-1-triggered ERK1/2 phosphorylation. **(A, B)** goat anti-mouse (GAM) and anti-NKG2D-stimulated NK cells were incubated with anti-DNAM-1 mAb or isotype control Ab followed by goat anti-mouse Ab for different lengths of times. Then, cells were fixed and permeabilized, stained with anti-pAKT (panel **A**) and anti-pERK1/2 (panel **B**) and analyzed by FACS. Results from 2 minutes of stimulation with anti-DNAM-1 mAb (filled histograms) or isotype control Ab (empty histograms) from one representative of three independent experiments is shown in the left panels. Quantitative changes in protein phosphorylation at the indicated lengths of time of three independent experiments (mean ± SD) are shown in the right panels. The ratio between the mean fluorescence intensity (MFI) of anti-DNAM-1-stimulated and Ctrl-Ig samples for each time point was used to calculate fold changes. Multiple t test was used for comparisons. *ρ < 0.05.

Primary cultured NK cells were stimulated for 18 hours with plate-bound anti-NKG2D mAb, recovered and restimulated with anti-DNAM-1 for different lengths of time. ERK1/2 and AKT phosphorylation were then evaluated by flow cytometry employing phosphospecific antibodies. Compared to control Ig, anti-DNAM-1 mAb was able to increase the levels of phosphorylated ERK1/2 and AKT (**Figure 4**). However, while AKT phosphorylation did not change significantly (**Figure 4A**), ERK1/2 phosphorylation was less pronounced in cells that were previously engaged with NKG2D (**Figure 4B**).These results suggest that persistent NKG2D stimulation limits the activation of specific signaling molecules after DNAM-1 engagement.

### 3.3 Receptor endocytosis is required for NKG2D-triggered DNAM-1 hypo-functionality

We have previously demonstrated that NKG2D binding with MICA is rapidly followed by the ubiquitin modification of the DAP10 intracellular Lysine that promotes both receptor internalization and lysosomal degradation (19, 20). Moreover, by employing the human NKL cell line transfected with wild-type DAP10 (DAP10WT NKL cells) or a mutated DAP10 protein in the intracellular Lysine (DAP10K84R), we have also shown that NKG2D ubiquitination and internalization are necessary for the activation of receptor-mediated intracellular signals and cytotoxicity (20).

Thus, we speculate that endocytosis-dependent signal transduction is required for the impairment of DNAM-1 functions.

**Figure 5:**
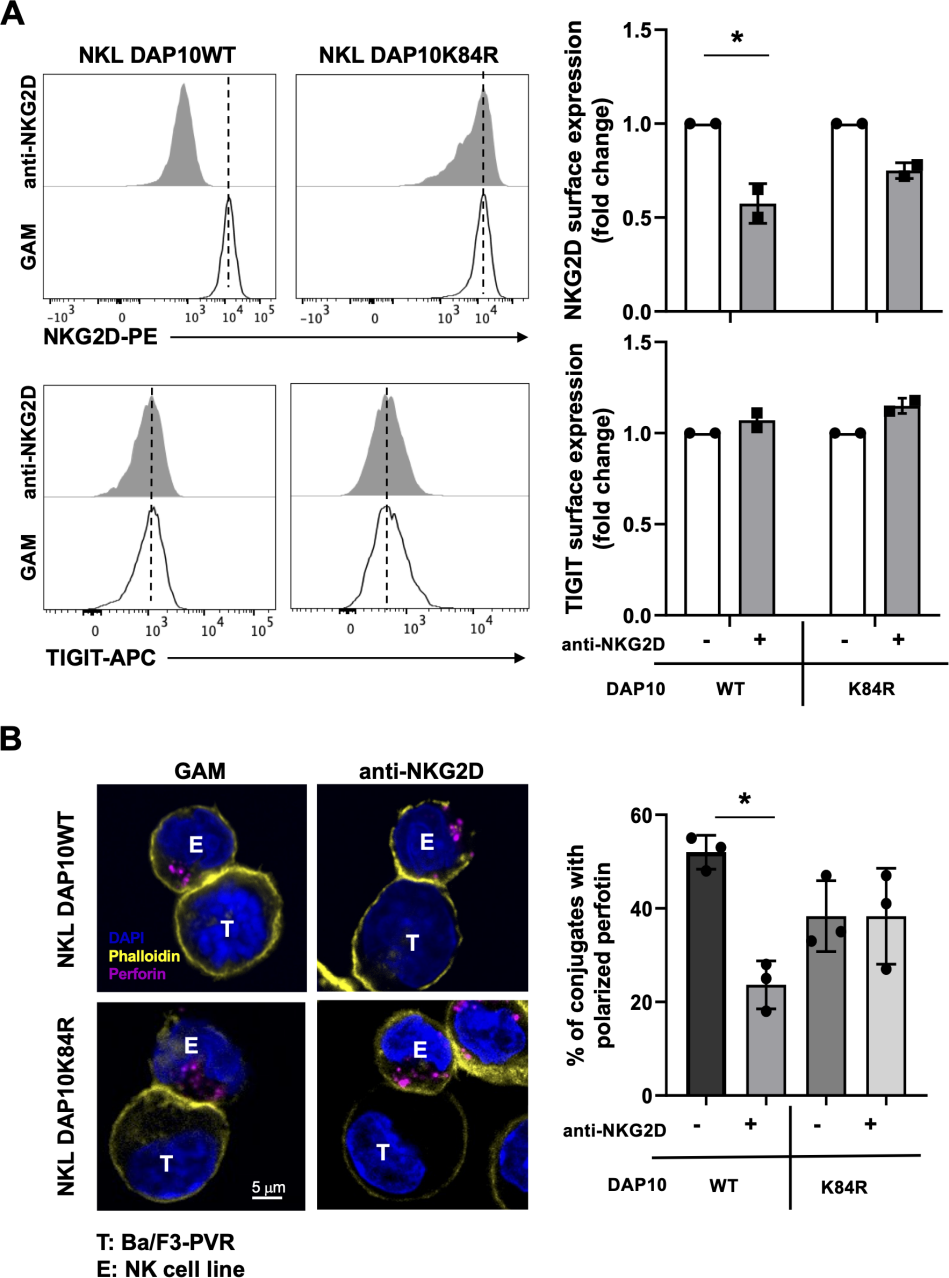
NKG2D internalization is required for impairment in DNAM-1-mediated cytotoxic granule polarization. **(A)** Surface expression of NKG2D (upper panels) and TIGIT (lower panels) receptors was evaluated on goat anti-mouse (GAM) or anti-NKG2D-stimulated DAP10 WT and DAP10K84R NKL. One representative of three independent experiments is shown in left panels. Data pooled from three independent experiments (mean ± SD) are shown in right panels (the mean fluorescence intensity, MFI value of GAM-stimulated cells in each experiment is set to 1). Paired t test was used for comparisons. *ρ < 0.05. **(B)** GAM and anti-NKG2D-stimulated DAP10 WT and DAP10K84R NKL (effector cells, E) were incubated with Ba/F3-PVR (target cells, T) for 30 minutes, fixed, permeabilized and stained with the anti-Perforin mAb followed by AlexaFluor594-conjugated GAM-IgG2b and AlexaFluor488-conjugated Phalloidin to visualize polarized actin. Cells were then counterstained with DAPI to visualize nuclei and acquired with LSM980 confocal microscope with a 63x/1.4 NA UPlanSAPO oil immersion objective (all from Zeiss). Right panel: Percentages of conjugates with polarized granules are shown as mean ± SD calculated on ≥30 conjugates for each NK cell type of three independent experiments. Paired t test was used for comparisons. *ρ < 0.05.

To test this hypothesis, we stimulated DAP10WT or DAP10K84R NKL cells with plate-bound anti-NKG2D mAb overnight. NKG2D and TIGIT expression was evaluated by flow cytometry. NKG2D internalization, evaluated as decreased surface expression, was partially impaired in the mutant respect to WT NKL cells, while TIGIT expression remains unaltered in both cell lines (**Figure 5A**).

NKL transfectants were stimulated overnight with anti-NKG2D mAb, recovered and cultured in the presence of PVR-expressing cells for 30 minutes. Their ability to polarize Perforin-positive lytic granule toward target cells was then analyzed by confocal microscopy. NKG2D stimulation significantly reduced the ability to polarize lytic granules in DAP10WT NKL cells (**Figure 5B**). However, granule polarization remained unaltered in DAP10K84R NKL transfectants, demonstrating that NKG2D internalization is required to induce DNAM-1 hypo-functionality. Western blot analysis demonstrates that Perforin and Granzyme B total levels do not decrease upon NKG2D stimulation in both cell lines (**Supplementary Figure S4**).

Taken together these results demonstrate that the impairment of DNAM-1-triggered signal transduction and cytotoxic granule release is a direct consequence of NKG2D endocytosis.

## 4 Discussion

NKG2D plays an important role in the eradication of cancers as underscored by experiments of antibody-mediated receptor neutralization (57) and by the phenotype of NKG2D deficient mice (58). Therefore, different NKG2D-based tools of cancer immunotherapy have emerged in the last few years (29).

A current approach consists in the use of multi-specific molecules simultaneously targeting tumor-associated antigen and NKG2D, thus eliciting NK cell-mediated cytotoxicity (59). For NKG2D binding, these recombinant molecules may both utilize domains of natural ligands (60–63) or single variable domain of anti-NKG2D antibodies (64–68). However, distinct modes of NKG2D aggregation may results in different outcomes in terms of cell activation as well as NKG2D-induced NK cell exhaustion.

In this paper, we demonstrate that NKG2D crosslinking mediated by the natural ligand MICA or an agonist anti-NKG2D antibody had different consequences on primary cultured human NK cells. Both stimuli provoked NK cell cytotoxic granule release and NKG2D internalization (**Figures 1A, B**) but only an overnight stimulation with plate-bound MICA increased TIGIT expression at protein and mRNA levels (**Figures 1C, D**).

Of note, our findings are in accord with previous data demonstrating that MICA and antibody-mediated NKG2D engagement are both able to induce target cell lysis but only the natural ligand triggers a full NK cell response through transcriptional events (69).

Our results also demonstrate that, without the requirement of a co-engaged activating receptor, NKG2D ligand stimulation is sufficient to increase TIGIT expression, further confirming a pivotal role for NKG2D/NKG2DL axis in driving NK cell hypo-functionality. However, whether TIGIT inhibits the signaling elicited by NK cell receptors other than DNAM-1 is still unknown.

Regarding the reason why TIGIT up-regulation only occurs upon ligand stimulation of NKG2D, it is likely that agonist antibody interacts with NKG2D in a different conformation and/or affinity compared to the natural ligand. Interestingly, a recent paper demonstrates that another agonist anti-NKG2D antibody, beside a comparable affinity with the natural ligand MICA, binds the receptor in larger epitopes and with a different stoichiometry, showing unique binding features (70). Thus, a plausible explanation is that agonist antibodies can elicit different signaling events inducing distinct conformational changes or affecting receptor translocation in membrane rafts.

Of note, a human antibody used to construct bispecific engagers (66, 68) resulted able to efficiently stimulate NK cell degranulation and block the interaction between NKG2D and its ligands (71). It could be interesting to analyze whether chronic NK cell stimulation with these molecules provokes desensitization of NKG2D and/or other unrelated receptors. A better understanding of the consequences of the distinct modalities of NKG2D engagement may contribute to the development of engager molecules able to stimulate NK cell activation without the induction of exhaustion.

Even though NKG2D crosslinking with plate-bound antibody is not able to trigger TIGIT up-regulation, a direct impairment of DNAM-1 activation has been observed. Indeed, DNAM-1 stimulation resulted defective in triggering ERK1/2 phosphorylation, in cytotoxic granule polarization and in promoting killing of PVR-positive target cells (**Figures 2**-**4**).

We have also provided evidence demonstrating that the negative regulation of DNAM-1 function is occurring upon NKG2D endocytosis (**Figure 5**). Of note, we have previously demonstrated that NKG2D localization in endosomes is required for a prolonged phosphorylation of ERK1/2 and the full activation of NK cell functions (20). All together our results support the conclusion that DNAM-1 impairment depends on NKG2D endosomal signaling.

It is also possible to hypothesize that upon sustained NKG2D stimulation and internalization, shared signaling molecules are sequestered in endosomal compartments and/or degraded together with the receptor complex and are no more available to transduce the subsequent DNAM-1-mediated biochemical signal.

In this context, previous data have provided evidence of multiple distinct activating receptors desensitized upon chronic NKG2D stimulation (44, 45). However, in these cases the role of NKG2D endocytosis has not been investigated.

All together our results contribute to shed light on the functional consequences of NKG2D engagement, demonstrating that a direct impact on DNAM-1-mediated signal transduction occurs independently from the modality of NKG2D crosslinking. The impairment of DNAM-1 activation may facilitate immune evasion of tumors expressing both NKG2D and DNAM-1 ligands. Moreover, a sustained NKG2D engagement may indirectly affect the outcome of immunotherapy since DNAM-1 has recently emerged as a key receptor that modulates patient’s response to checkpoint receptor inhibition (72–76). Therefore, a deeper knowledge of the molecular mechanisms underlying NKG2D/DNAM-interplay may help to the development of more efficient therapy to block the checkpoint receptors.

Understanding the molecular mechanisms responsible for evasion of NK cell surveillance in TME may help the development of strategies aimed to prevent NK cell exhaustion or with the potential to reinvigorate dysfunctional cytotoxic activity.

## Data Availability

The original contributions presented in the study are included in the article/**Supplementary Material**. Further inquiries can be directed to the corresponding authors.
