# Supplementary material for: NKG2D triggering hampers DNAM-1-mediated signaling in human NK cells
Source: Front Immunol. 2025 May 12;16:1575059. doi: 10.3389/fimmu.2025.1575059 (PMC12104298; doi:10.3389/fimmu.2025.1575059)
Supplement: Supplementary file 1 [file DataSheet1.docx]

Supplementary Material

**
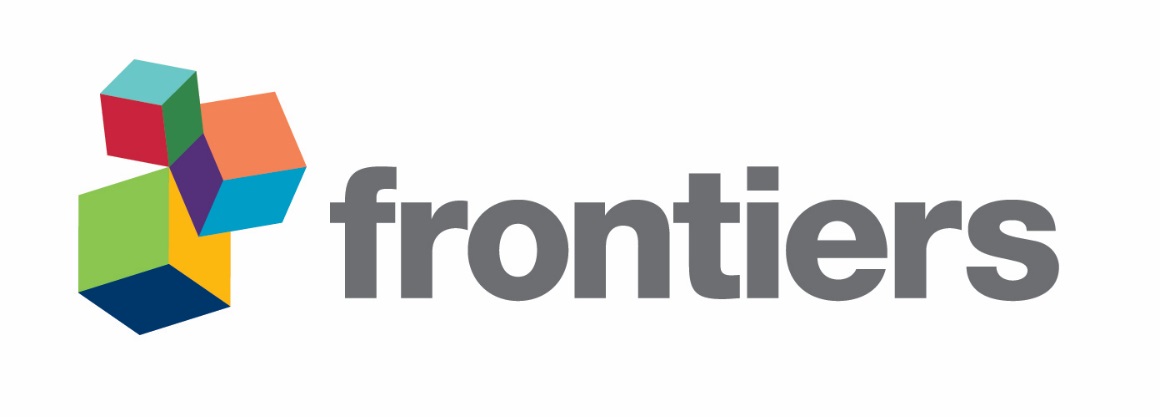
**


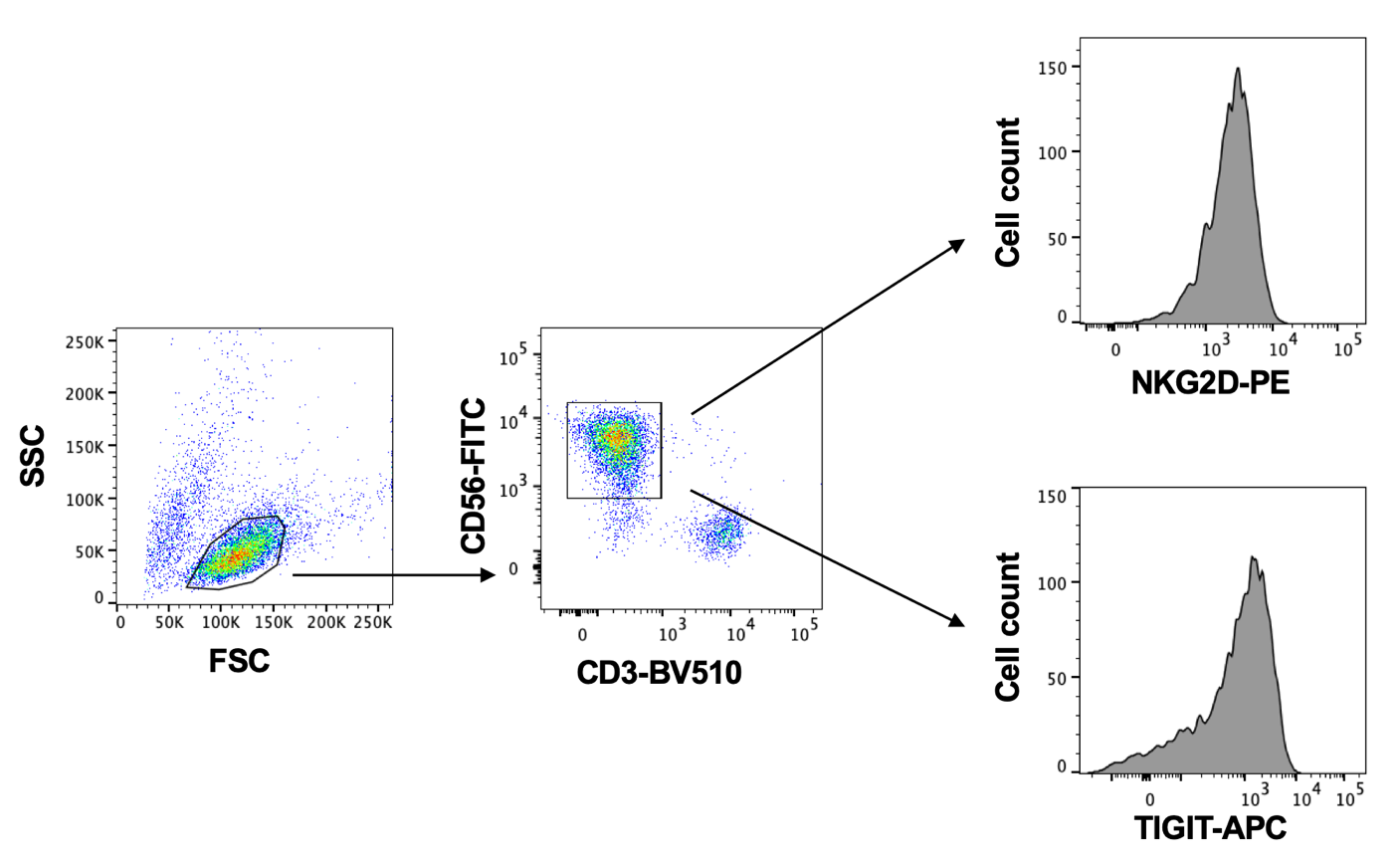


**Supplementary Figure 1:** **Gating strategy used in flow cytometry experiments:** Live cells were gated using morphologic parameters and NK cells were gated as CD56^+^ CD3^-^ cells. Membrane receptor expression was evaluated on NK cells.


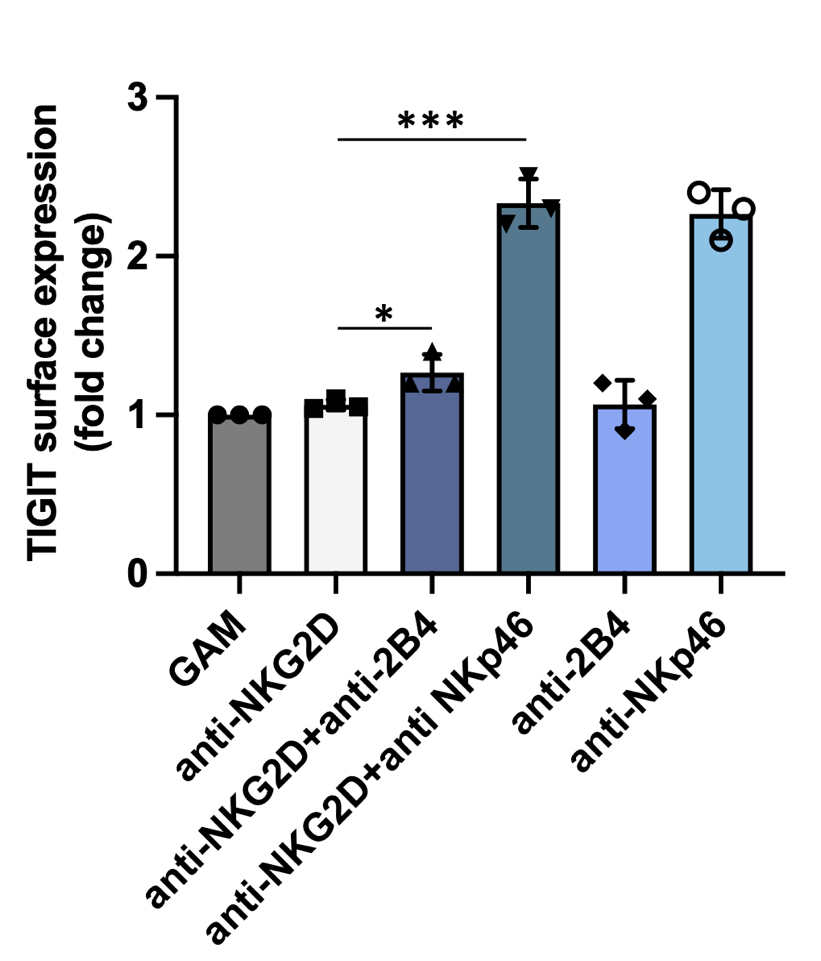


**Supplementary Figure 2:** **Stimulation with anti-NKp46 up-regulates TIGIT expression**

TIGIT surface expression was evaluated on unstimulated, anti-NKG2D, anti-NKG2D+anti-2B4, anti-NKG2D+anti-NKp46, anti-2B4 and anti-NKp46-stimulated NK cells by FACS analysis. Data pooled from three independent experiments (mean ± SD) are shown (the MFI value of GAM-stimulated NK cells in each experiment is set to 1). One-way ANOVA was used for comparisons. *Ρ < 0.05, ***Ρ < 0.001.


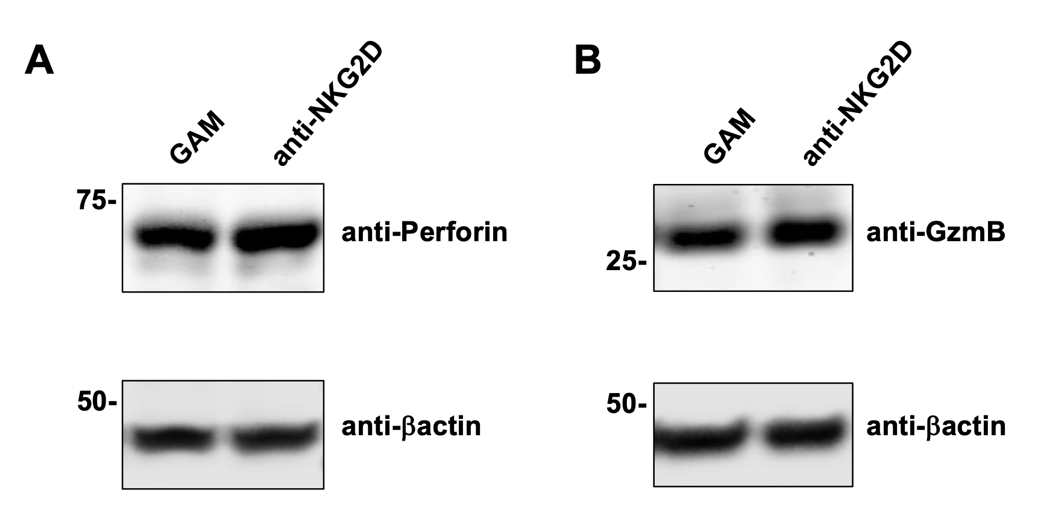


**Supplementary Figure 3:** **Stimulation with anti-NKG2D does not alter Perforin and Granzyme B total expression on primary human NK cells**

Goat anti-mouse (GAM) and anti-NKG2D-stimulated NK cells were lysed and separated on 8% (A) or 12% (B) SDS-PAGE. Proteins were transferred on nitrocellulose membrane and incubated with the indicated antibodies. One representative experiment is shown.


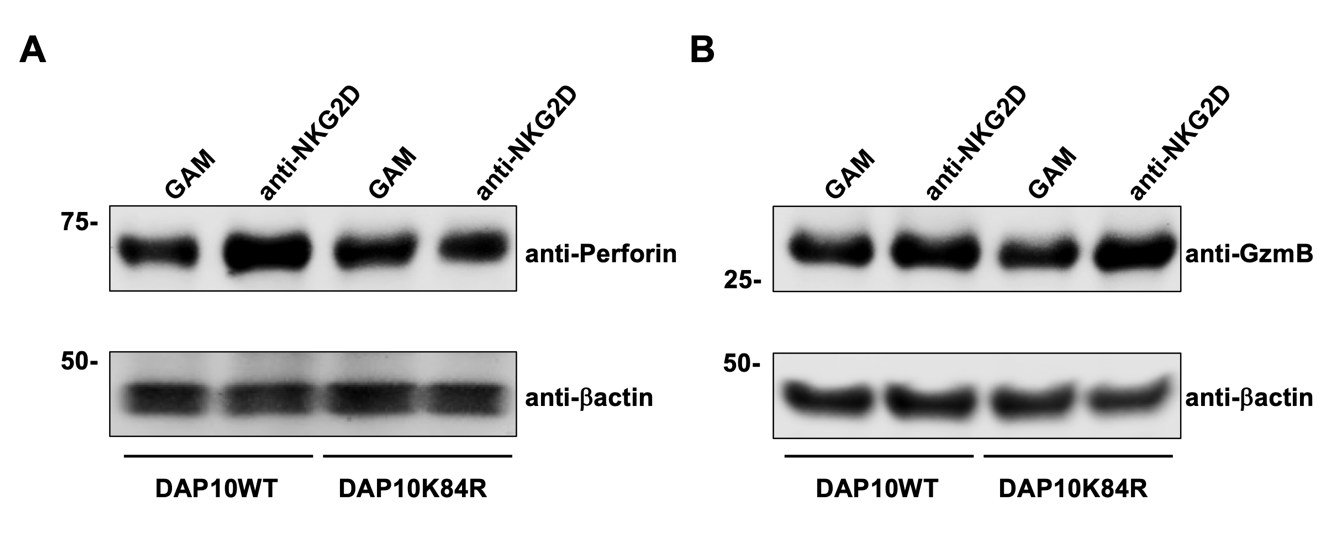


**Supplementary Figure 4:** **Stimulation with anti-NKG2D does not alter Perforin and GranzymeB total expression on NKL transfectants**

Goat anti-mouse (GAM) and anti-NKG2D-stimulated DAP10WTNKL and DAP10K48RNKL cells were lysed and separated on 8% (A) or 12% (B) SDS-PAGE. Proteins were transferred on nitrocellulose membrane and incubated with the indicated antibodies. One representative experiment is shown.
